# Stress Dependence on Relaxation of Deformation Induced by Laser Spot Heating

**DOI:** 10.3390/ma15186330

**Published:** 2022-09-13

**Authors:** Yuma Murata, Tomohiro Sasaki, Sanichiro Yoshida

**Affiliations:** 1 Graduate School of Science and Technology, Niigata University, Niigata 9502181, Japan; 2 Department of Chemistry and Physics, Southeastern Louisiana University, Hammond, LA 70401, USA

**Keywords:** non-destructive testing, optical interferometry, residual stress, thermal expansion, elasticity

## Abstract

This paper deals with a non-destructive analysis of residual stress through the visualization of deformation behaviors induced by a local spot heating. Deformation was applied to the surface of an aluminum alloy with an infrared spot laser. The heating process is non-contact, and the applied strain is reversible in the range of room temperature to approximately +10 °C. The specimen was initially pulled up to elastic tensile stress using a tensile test machine under the assumption that the material was subject to the tensile residual stress. The relaxation behaviors of the applied strain under tensile stress conditions were evaluated using contact and non-contact methods, i.e., two strain gauges (the contact method) and a two-dimensional electronic speckle pattern interferometer (non-contact method). The results are discussed based on the stress dependencies of the thermal expansion coefficient and the elasticity of the materials.

## 1. Introduction

Residual stress is induced by various manufacturing processes such as machining, welding processes, and heat treatment. In particular, tensile residual stress causes a reduction in fatigue strength, and stress corrosion cracking. Residual stress analysis is still a subject of investigation. There are several commonly used techniques to measure residual stress destructively. The principle of these techniques is the measurement of strain released by machining processes including hole drilling and cutting with electric discharge machining. The released strain can be measured using strain sensors in contact or non-contact ways. Optical measurement techniques such as electronic speckle pattern interferometry (ESPI) [1,2], holographic interferometry [3,4], moiré interferometry [5,6], and digital image correlation (DIC) [7,8] are known as non-contact and full-field strain measurement techniques. These techniques allow the full-field measurement of residual stress in combination with the destructive way, while they involve an irreversible process. Recently, a method using microfabrication with a pulsed spot laser and digital holography has been proposed [9]. This method applies minimal destruction in a hole diameter of 20 μm. In addition, an attempt has been made to measure the released strain by annealing with laser spot heating [10]. On the other hand, diffractometry using neutrons [11] and X-rays [12] is known as the most common way of conducting non-destructive residual stress analysis. This method measures the stress through the change in the lattice constant. In addition, measurements of electromagnetic waves [13] and acoustic waves [14] using contact probes are often included in the non-destructive techniques. These non-destructive techniques have a limitation in the measurement area of several hundred mm^2^ per measurement, leading to a time-consuming process to measure a wide range of residual stresses. A technique equipped with non-destructive and non-contact measurements is not available.

Previously, we made attempts to evaluate residual stress through the visualization of reversible deformation with optical techniques. The study focused on the stress dependence of elasticity using the same principle as the acoustic elasticity and showed that the elastic deformation behavior applied with a tensile machine depends on the applied stress [15,16]. More recently, a study [17] investigated thermal deformation behavior under the stressed condition, focusing on the stress dependence of thermal characteristics. Both properties of elasticity and thermal expansion have stress dependency derived from the non-linearity and anharmonicity of the interatomic potential [18,19,20]. These studies suggested that residual stress can be estimated through the visualization of reversible thermal deformation in the temperature range of ±10 °C. However, the above methods need devices to apply reversible deformation to the material, and the possibility of a non-contact method still remains an issue.

The present study investigated the local thermal deformation behavior induced by spot heating. A heating laser beam was applied to a local area of a residually stressed specimen for a short period of time. This heating process is non-contact, and the induced temperature change is reversible in the range of room temperature to approximately +10 °C. The resultant thermal strain was measured with contact and non-contact methods. For the contact method, strain gauges were used, and for the non-contact method, an ESPI setup was used. It was expected that the thermal deformation behavior would be affected by the stress field due to the stress dependence of thermal expansion. At the same time, the local heating under the stressed condition was expected to cause strain relaxation in the surrounding area, resulting in elastic relaxation. The possibility for non-destructive residual stress analysis is discussed through the visualization of the reversible deformation behavior in the heated and surrounding areas.

## 2. Materials and Methods

### 2.1. Specimen

**Figure 1:**
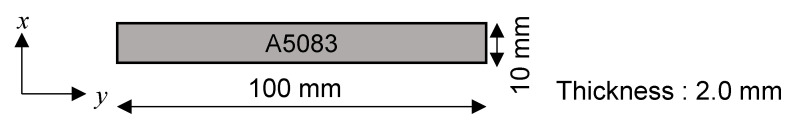
Test specimen.

An industrial aluminum alloy (ISO AA5083) sheet with a thickness of 2.0 mm was used in this study. Table 1 shows the standard chemical composition of the AA5083 alloy. This alloy is a solid-solution-hardened Al-Mg-based alloy. Although the temperature change applied in this study is small enough in the range of approximately 10 °C, the alloy is microstructurally less sensitive to the heating process. The modulus of longitudinal elasticity of this material is 70 GPa, and the 0.2% proof stress is approximately 145 MPa. The sheet was cut into specimens with a length of 100 mm and a width of 10 mm by electrical discharge machining, as shown in Figure 1. The specimens were annealed at 300 °C in air for three hours to remove the as-received residual stress. The specimens used for the visualization of the strain field using speckle pattern interferometry were coated with a white lacquer spray, and those used for strain gauges were polished with #800 emery abrasive paper.

### 2.2. Local Spot Heating

**Figure 2:**
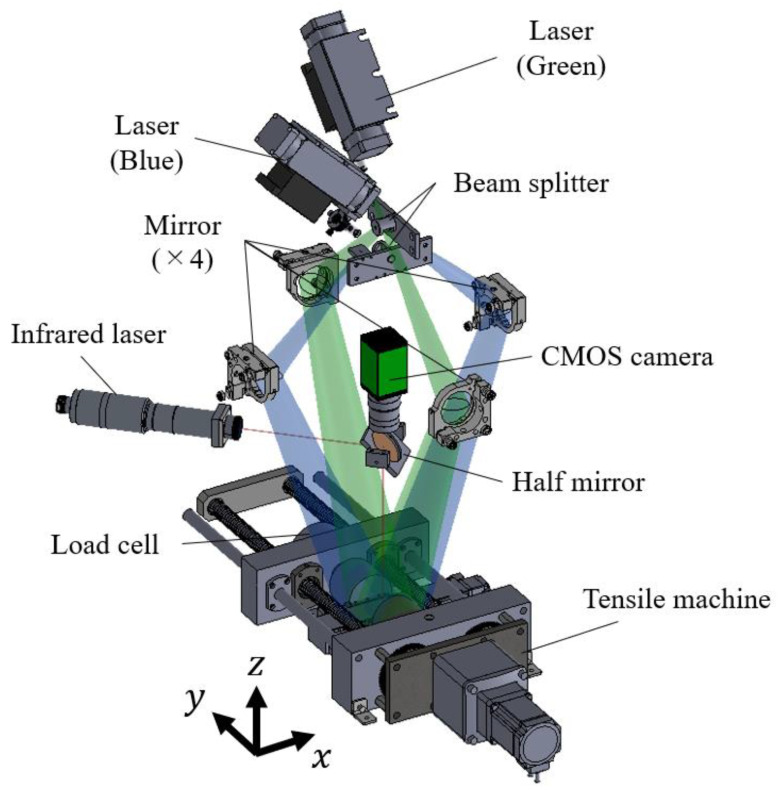
Optical setup and devices for local spot heating test under the stressed condition.

Figure 2 shows a setup for local spot heating under tensile stress conditions, and a two-dimensional electronic speckle pattern interferometer (2D-ESPI) used for the visualization of thermal deformation, as described in the previous section. A tensile test machine was used to apply an external load to the specimen under the assumption that the material was subject to the tensile residual stress. The specimen was initially pulled up to tensile stress levels (initial stress, $\sigma_{i}$) at a crosshead speed of 0.002 mm/s, measuring the external load with a load cell attached to the tensile machine (KYOWA ELECTRONIC INSTRUMENTS CO., LTD, LUA-A). The initial stress condition ranged from 0 to 120 MPa, which is approximately 80% of the proof stress at maximum. The condition of initial stress $\sigma_{i}=0$ MPa indicates that one end of the specimen was fixed with a clamp and the other end was free. Under each initial stress condition, the specimen was subjected to spot heating using an infrared laser (SPI Lasers red ENERGY G4, wavelength of 1064 nm). The infrared laser was irradiated perpendicularly to the surface of the specimen at an angle of 45° to the tensile axis through a half mirror, as shown in Figure 2. The spot diameter was adjusted to 1 mm by a focusing lens via a half mirror.

**Figure 3:**
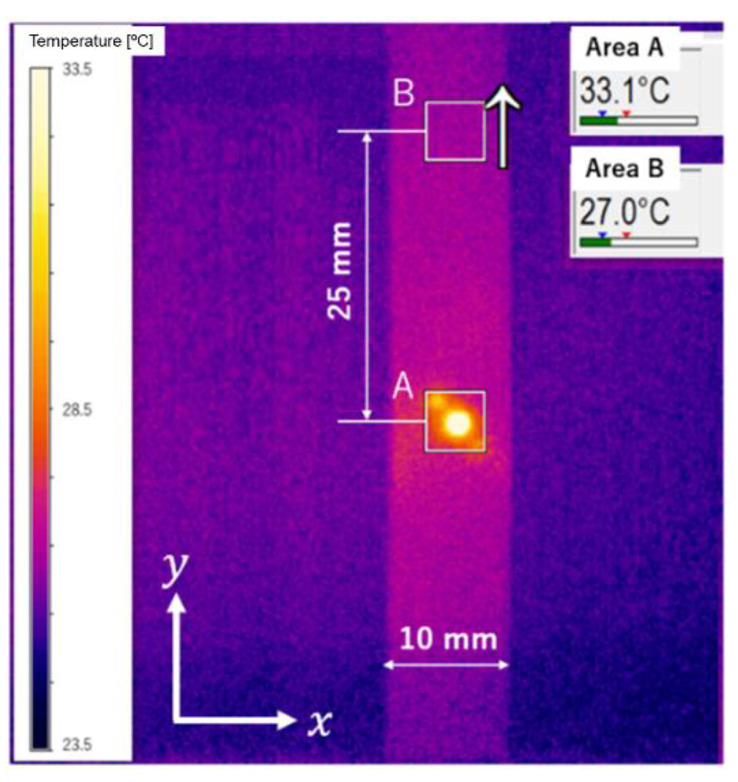
Thermograph of a test specimen during local heating.

**Figure 4:**
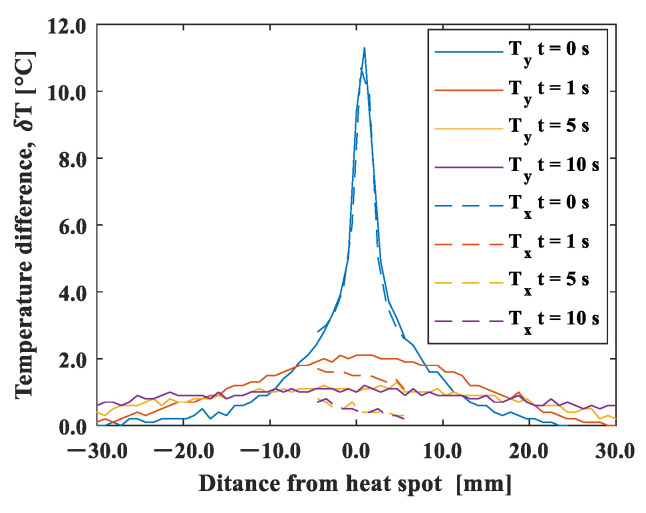
Temperature distribution around the heat spot.

Figure 3 shows a typical example of a thermal image taken using an infrared camera (Optris GmbH, PI80). The temperature distribution around the heat spot is shown in Figure 4. The temperature values are plotted in the x- and y-directions as $T_{x}$ and $T_{y}$, respectively. The initial profile at t = 0 exhibited a Gaussian distribution, and it was symmetric for the other directions. The maximum temperature increase at the heating point was about 10 °C from room temperature. 

**Figure 5:**
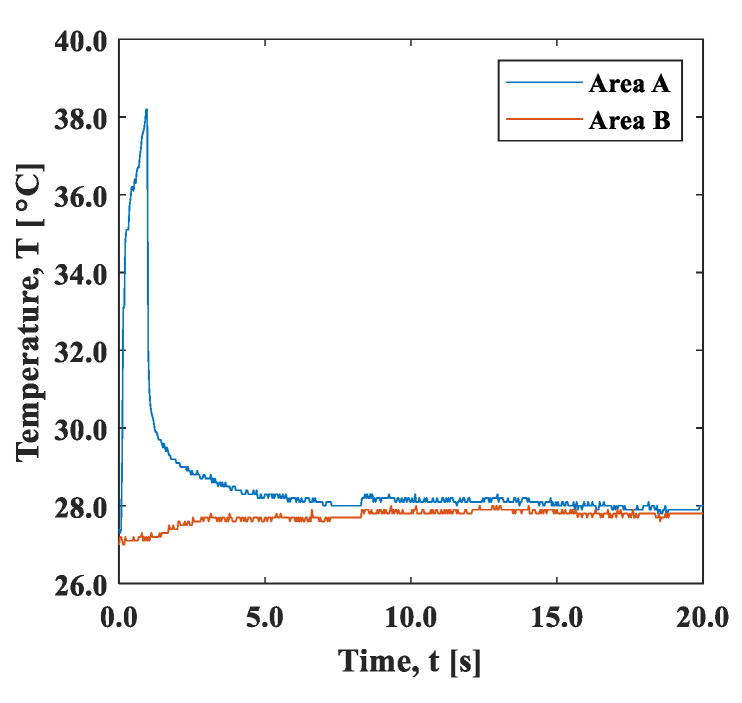
Time variation of temperature of areas A and B shown in Figure 3.

Figure 5 shows the time variation of the average temperature in the 5 × 5 mm^2^ areas shown by squares A and B in Figure 3. Heat diffusion occurred simultaneously with the local spot heating for 1 s. The result shows that the average temperature in area A during heating reached up to 38 °C in about one second after the start of laser irradiation, and then it decreased rapidly after the heating stopped. Meanwhile, in area B, which was 25 mm away from the center of the spot, the temperature difference was less than 1 °C, indicating that the effect of heating was negligible in this area.

### 2.3. Strain Measurement

The thermal deformation behavior during the local spot heating was evaluated using two devices; namely, a strain gauge and a 2D-ESPI. The strain gauge measurement aimed to accurately measure the thermal strain to support the following 2D-ESPI. The capability of an in-plane and non-contact evaluation of residual stress will be discussed in the ESPI section.

(i) Strain gauge

**Figure 6:**
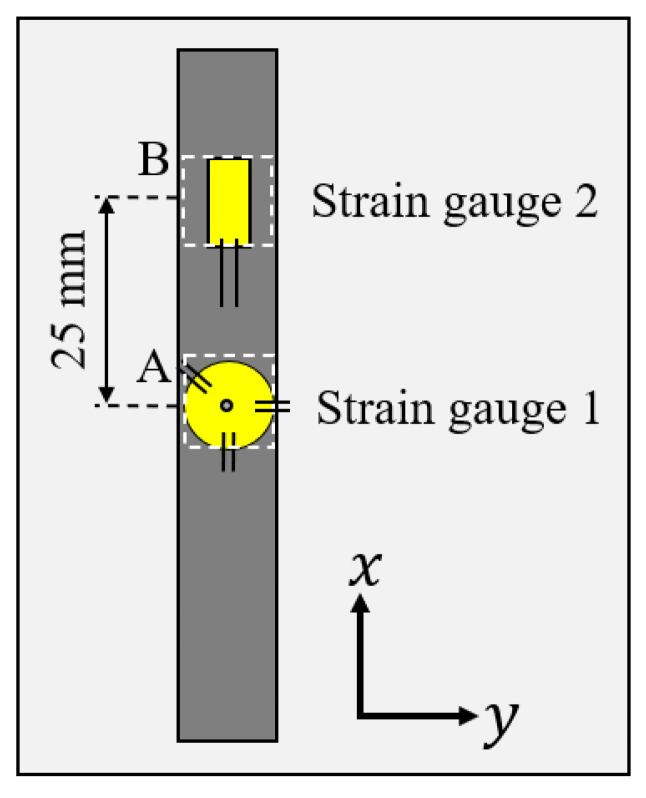
Strain gauge mounting position.

Strain values around the local spot-heated area (area A in Figure 3) were measured using a rosette strain gauge with a central hole diameter of 2 mm and a gauge length of 1.5 mm, as shown in Figure 6. At the same time, the strain in area B, which was 25 mm away from the heat spot, was measured using a single-axis strain gauge with a gauge length of 5.0 mm. The resistance of both strain gauges was 120 ohms.

(ii) Two-dimensional electronic speckle pattern interferometry (ESPI)

**Figure 7:**
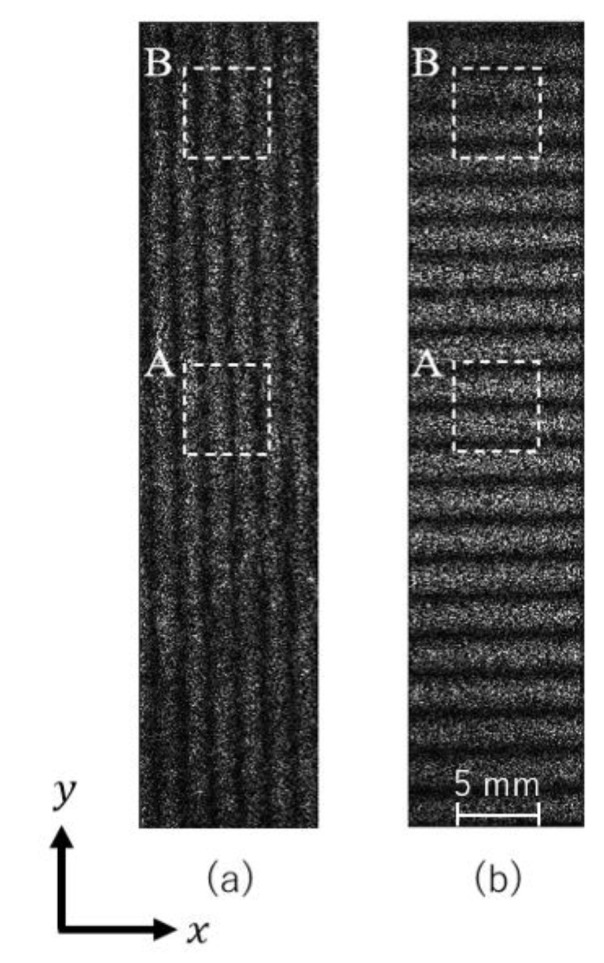
Carrier fringe image: (**a**) x-direction; (**b**) y-direction.

Two optical systems sensitive to a single-axis displacement were arranged in the tensile direction (y-direction) and transverse to the tensile direction (x-direction), as shown in Figure 2. Each system was based on the “Dual-beam ESPI” sensitive to in-plane displacement [15,16,17]. A blue laser with a wavelength of 472.9 nm and a green laser with a wavelength of 532.3 nm (LASOS Lasertechnik GmbH, DPSS laser) were used for the light sources in the y- and x-directions, respectively. The laser beam expanded by the expander was split into two paths by a splitter. The split laser beams irradiated the surface of the specimen at the same incident angle of 18.83° through mirrors. The speckle intensity reflecting the surface changed depending on the displacement in the sensitive direction due to the optical path difference between the two interferometric arms. A prism spectroscopic 3CMOS camera (JAI Corporation, AP-1600T) was used to capture the speckles generated by the two light sources. The frame rate and resolution were 60 fps and 1280 × 960 pixels, respectively. The speckle patterns that originated from the two-color beams were split using a prism inside the camera [21]. The change in speckle intensity during the local spot heating test was calculated numerically by subtracting each frame of the image from a later frame taken after thermal expansion. The interferometric fringe patterns representing the displacement field can be obtained for each light source. Displacement u,v  in the x,y-directions when the number of fringes increases by $n_{x},n_{y}$ can be given by

(1) $$\left[\begin{array}{c} u\\ v \end{array}\right]=\left[\begin{array}{c} \frac{\lambda_{x}n_{x}}{2\sin\theta }\\ \frac{\lambda_{y}n_{y}}{2\sin\theta } \end{array}\right]$$

where $\lambda_{x}$ and $\lambda_{y}$ are the wavelengths of the light sources for the two optical configurations, and θ is the incident angle. The angle of θ was 18.83°. By calculating the displacement in the x- and y-directions independently, it is possible to measure the two-dimensional in-plane displacement distribution. In addition, carrier fringes were introduced by rotating the mirror, as employed in the previous study [17]. Figure 7 shows an example of a carrier fringe image. A fringe pattern with a constant interval was artificially introduced by aligning the mirror on one optical arm. This makes the fringe analysis based on the spatial frequency easier. The actual displacement field was obtained by subtracting the displacement from the carrier from the displacement of a given image. Approximately four carrier fringes per 10 mm were introduced in the tensile direction. The strain field can be obtained through the following process.

**Figure 8:**
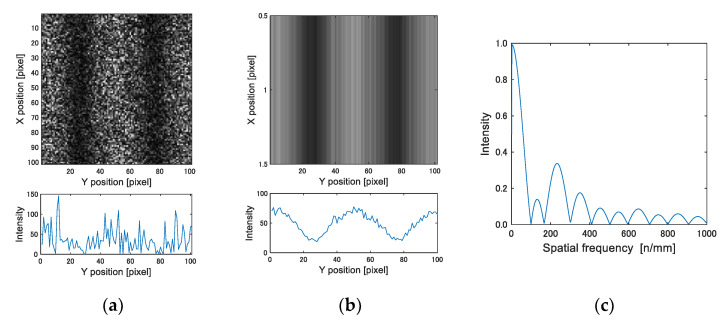
Fringe image analysis by FFT: (**a**) fringe image and intensity profile; (**b**) averaged intensity profile; and (**c**) FFT spectrum.

Figure 8a shows a cropped image of a 5 × 5 area (101 × 101 pixels) in Figure 7b. An intensity distribution, as shown in Figure 8b, is obtained by averaging intensity values in the x-direction. Since the number of fringes increases with the difference in the displacement, the spatial frequency of the speckle intensity represents the strain level. The Fourier spectrum computed from the intensity distribution is shown in Figure 8c. To increase the resolution of the frequency analysis, the sampling points of the intensity profile were interpolated to 100 times the original plot data. The peak frequency near the frequency of the carrier, from +200 to −100 around 200 n/mm, was searched in the spectrum. The peak frequency indicates the number of fringes n per unit length. The mean strain at the center coordinate of the cropped area was determined from the number of fringes using Equation (2).

(2) $$\varepsilon =\left[\begin{array}{c} \varepsilon_{xx}\;\;\varepsilon_{xy}\\ \varepsilon_{yx}\;\;\varepsilon_{yy} \end{array}\right]=\left[\begin{array}{cc} \frac{\partial u}{\partial x} & \frac{1}{2}\left(\frac{\partial u}{\partial y}+\frac{\partial v}{\partial x}\right)\\ \frac{1}{2}\left(\frac{\partial v}{\partial x}+\frac{\partial u}{\partial y}\right) & \frac{\partial v}{\partial y} \end{array}\right]≅\left[\begin{array}{cc} \frac{u}{l_{x}} & \frac{1}{2}\left(\frac{u}{l_{y}}+\frac{v}{l_{x}}\right)\\ \frac{1}{2}\left(\frac{v}{l_{x}}+\frac{u}{l_{y}}\right) & \frac{v}{l_{y}} \end{array}\right]$$

where $l_{x},\;l_{y}$ are the length of the cropped area, and u,v are the displacements in the x- and y-directions in the cropped area, respectively. The strain distribution was obtained by shifting the cropped area by one pixel in the sensitive directions.

## 3. Results and Discussion

### 3.1. Relaxation Behavior of Strain and Temperature

It is difficult to measure temperature and strain changes during heating because of the rapid and unstable temperature change. Thus, we focused on the cooling process after the laser spot heating. The thermal diffusion in the cooling process can be discussed based on an instantaneous heat source applied to the surface. The equation of heat conduction is generally expressed by

(3) $$\frac{\partial T\left(x,y,z,t\right)}{\partial t}\frac{1}{K}=\left(\frac{\partial^{2}T}{\partial x^{2}}+\frac{\partial^{2}T}{\partial y^{2}}+\frac{\partial^{2}T}{\partial z^{2}}\right)$$

where T(x,y,z,t) is the temperature, K=k/ρc is the thermal diffusivity, and *k,* ρ, and c are the thermal conductivity, the density, and the thermal capacity, respectively. When an initial temperature distribution at *t* = 0 is given by a function of coordinates as $T_{0}\left(x,y,z\right)$, Equation (1) is satisfied by the following solution [22]:

(4) $$T\left(x,y,z,\;t\right)=\frac{1}{{\left(\sqrt{4\pi Kt}\right)}^{3}}\int_{-\infty }^{\infty }\int_{-\infty }^{\infty }\int_{-\infty }^{\infty }T_{0}\left(x^{\prime },y^{\prime },z\prime \right)\exp\left\{-\frac{{\left(x^{\prime }-x\right)}^{2}+{\left(y^{\prime }-y\right)}^{2}+{\left(z^{\prime }-z\right)}^{2}}{4Kt}\right\}dx\prime dy\prime dz\prime$$

When a point heat source is instantaneously applied at point (0,0,0), at *t = 0*, the initial temperature is expressed by

(5) $$T_{0}\left(x,y,z,0\right)=\delta \left(x\right)\delta \left(y\right)\delta \left(z\right)$$

where δ(x) is the Dirac delta function. For heat conduction on the surface of a semi-infinite body, where the point heat source is applied to the surface (z=0) in the x−y plane, the solution to Equation (3) is given by

(6) $$T\left(x,y,z,t\right)=\frac{2Q_{0}}{\rho c{\left(\sqrt{4\pi Kt}\right)}^{n}}\exp\left\{-\frac{x^{2}+y^{2}+z^{2}}{4Kt}\right\}$$

where $Q_{0}\;$ is the volumetric heat source applied to the initial point, and *n* represents the spatial dimension of heat diffusion. We set the time t in Equation (6) as follows. In the experiment, we set the origin of time ($t_{experiment}=0)$ to the moment when the heating source was removed. To evaluate Equation (6), we used a finite time step to express the instantaneous heating. These operations required us to adjust the origin of t so that the spatial temperature profile resulting from Equation (6) was consistent with the experimental counterpart. We calibrated t  so that Equation  (6)  yielded the temperature profile closest to the experimental counterpart.

**Figure 9:**
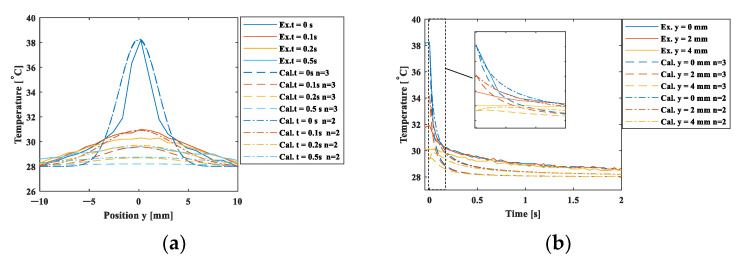
Comparison of the cooling process in the experiment and the point heat source model. (**a**) spatial changes; (**b**) temporal changes.

Figure 9 shows the temperature obtained by Equation (6) for the position (0,y,0) plotted on the experimental value measured by the infrared camera. The calculation was conducted for the spatial dimensions *n* = 2 and 3. The spatial and temporal changes are shown in Figure 9a,b, respectively. The calculated values shown by the dashed and chained lines (“Cal.”) are roughly consistent with the measured value “Ex.” in the shorter time within 0.1s, while larger temperature drops are exhibited. This is attributed to the fact that the thermal model assumes the heat diffusion in the semi-infinite body. The actual heat flow may be slower in the longer time due to the heat conduction reaching the end of the specimen. In addition, the spatial dimension *n* = 2 is in better agreement, implying a dominant heat flow in the two-dimensional diffusion in the *x-y* plane. 

**Figure 10:**
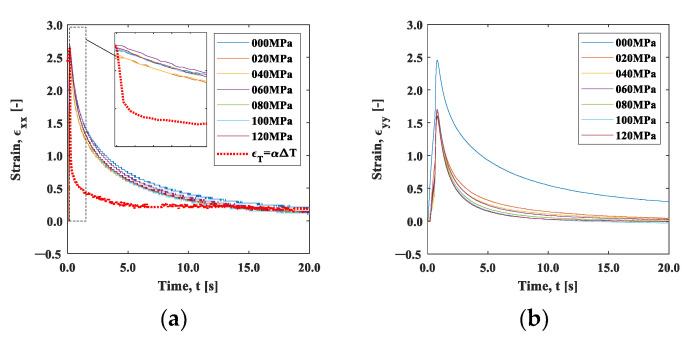
Strain–time curve measured by a strain gauge: (**a**) x-direction; and (**b**) y-direction.

On the other hand, Figure 10 shows the strain change around the heating point (area A). In the x-direction (Figure 10a), the maximum strain of approximately 2.5 $\times 10^{-4}$ was roughly consistent with the linear expansion per 10 °C, and it showed no significant difference in the initial tensile stress condition of 0 to 120 MPa. The maximum strain $\varepsilon_{yy}$ in the y-direction (Figure 10b) was almost equal to $\varepsilon_{xx}$ at the initial stress of 0 MPa, while under the initial tensile stress condition, it obviously became smaller. This result indicates that the thermal expansion was hindered due to the constraint in the y-direction. The thermal expansion coefficient α can be regarded as a constant in the temperature change within 10 °C. According to the data of the aluminum alloy obtained by Takeuchi et al., the change in the thermal expansion coefficient α in this temperature range is on the order of 10^−8^ [K^−1^] [23]. The thermal strain in area A was estimated as $\epsilon_{T}=\alpha \Delta T$, using the temperature change measured with the infrared camera shown in Figure 9a. Here, the temperature difference between the positions x=0 mm and x=2.5 mm was used for ΔT and 2.38 $\times \;10^{-5}$ was used for the thermal expansion coefficient, α. The estimated thermal strain is plotted as a function of time in Figure 10a by a dotted line. The estimated strain value deviated from the value measured with the strain gauge; the estimated thermal strain exhibited a more rapid decrease than the measured strain. The measured strain includes the thermal strain and the elastic strain due to the constraint of the surrounding area. When the thermal strain was instantaneously removed by heat diffusion in the cooling process, elastic strain relaxation subsequently occurred. The rapid drop in the thermal strain $\epsilon_{T}$ shown in Figure 10a implies that the elastic strain relaxation was independent of the heat diffusion process discussed in Figure 9. The disagreement between the temperature measurement and strain measurement rules out the role of thermal strain in the observed strain measurement. 

**Figure 11:**
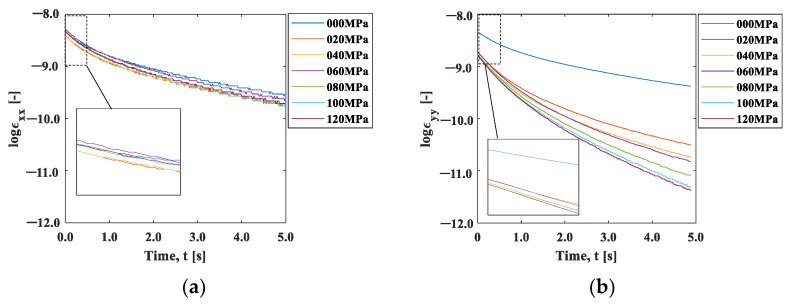
Log (strain)–time curve: (**a**) x-direction; and (**b**) y-direction.

We now discuss the observed strain relaxation behavior. It is well known that viscoelastic materials exhibit exponential strain relaxation due to their viscosity. Although the origin of viscosity in this experiment is unclear, the strain relaxations shown in Figure 10 indicate exponential-like decay. Figure 11a,b are natural logarithm plots of the strain change during the cooling after the maximum temperature in Figure 10. It can be seen that the slope is not completely linear, indicating that the exponential-like decay cannot be represented by a single decay constant. However, a close look at the initial part of the graph indicates that the trend between *t* = 0 s and *t* = 0.4 s is approximately linear, as the inserts in these figures exhibit. 

**Figure 12:**
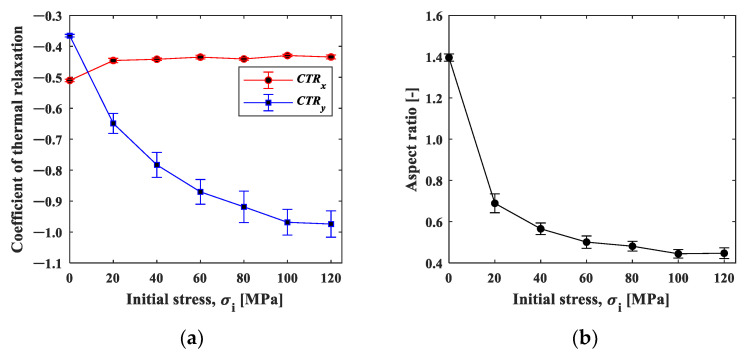
(**a**) *CSR*–initial stress curve plot; and (**b**) aspect ratio of *CSR_x_/CSR_y_*.

We evaluated this initial slope, called the coefficient of strain relaxation (*CSR*), by least squares fitting the natural logarithmic curve within 0.4 s in Figure 11. Figure 12a shows the results plotted as a function of the initial stress condition. The $CSR_{y}$ decreased significantly with the initial stress, indicating that the relaxation occurred faster with the increase in the initial stress. In contrast, the $CSR_{x}$ showed a slight increase with the initial strain, indicating that the strain relaxation behavior was opposite to $CSR_{y}$. The elastic modulus has stress dependency originating from the non-linearity and anharmonicity of the interatomic potential [18]. It is known that the elasticity decreases under tensile stress due to the fact that the slope of the potential curve decreases as the interatomic distance increases from its equilibrium position. It should be noted that the condition $\sigma_{i}=0$ is essentially different from the other initial stress conditions because one end of the specimen is under no constraint. The difference in the stress dependence between $CSR_{x}$ and $CSR_{x}\;$ observed in Figure 12a can be ascribed to Poisson’s effect. The tensile stress in the *y*-direction leads to the compressive stress in the *x*-direction. 

Figure 12b shows the aspect ratio, *CSR_x_/CSR_y_*_._ The aspect ratio under the initial stress $\sigma_{i}=0$ MPa was about 1.4, indicating that larger shrinkage occurred in the x-direction. This may be due to the fact that the deformation was constrained in the x-direction with the clamp. The aspect ratio decreased with the increase in the initial stress, and then it approached Poisson’s ratio in elasticity. This fact supports our proposition that the influence of elasticity, not the thermal effect, is dominant in the cooling process.

**Figure 13:**
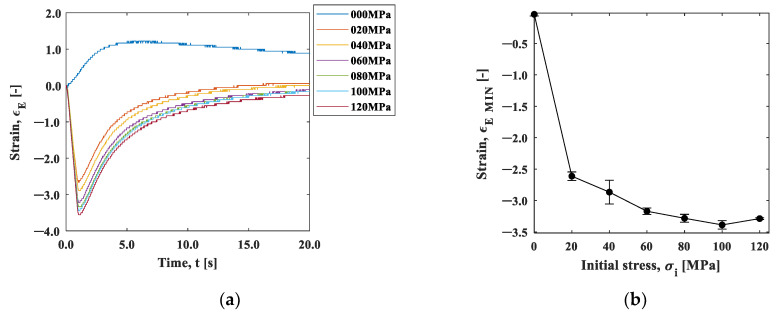
(**a**) Strain–time curve; and (**b**) strain–initial stress curve.

Figure 13a shows the strain change in area B, 25 mm away from the heat spot. The strain changed in the negative direction (compressive direction) in the initial tensile stress conditions, except for the condition of 0 MPa. As shown in Figure 5, the temperature difference in area B was within +1 °C, and the thermal effect was small enough to neglect. This result indicates that the elastic strain was recovered by the thermal expansion of the spot-heated area. The change in strain may follow the exponential function, and this also applies to area A. Figure 13b shows the minimum strain in the strain–time curve plotted against the initial stress. The minimum strain decreased depending on the initial stress, demonstrating the stress dependence of the compressive strain relaxation.

**Figure 14:**
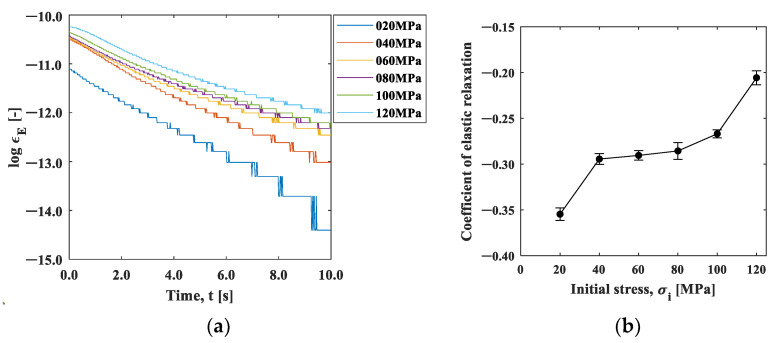
(**a**) Log (strain)–time curve; and (**b**) CER–initial stress curve.

**Figure 15:**
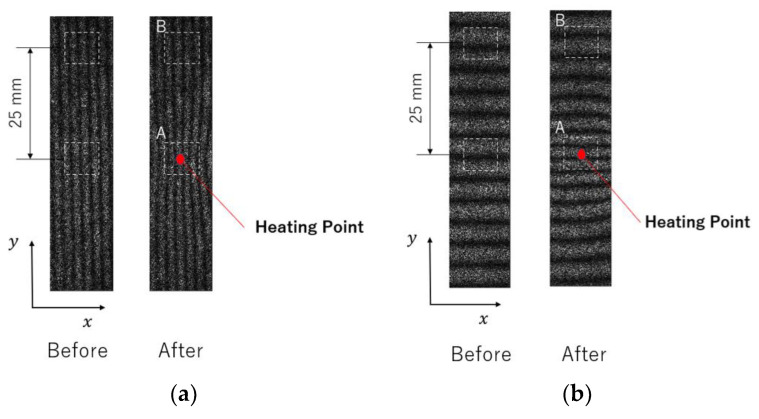
Fringe images before and after the spot heating, in the (**a**) x-direction and (**b**) y-direction.

Figure 14a shows the natural logarithm of the strain after the minimum strain at the initial stress of 20–120 MPa. All stresses are shown linearly; therefore, Figure 15b shows the slope of the line up to 1 s in Figure 14a as the coefficient of elastic relaxation (CER). Figure 14b shows that the *CER* increased with the increase in the initial stress. In other words, the rate of elastic relaxation strain became slower due to the decrease in the elastic modulus with increasing stress. As shown in Figure 12, the rate of thermal shrinkage of the heated area (area A) was faster in the y-direction. In contrast, the elastic relaxation of the less heat-affected area (area B) shown in Figure 14 increased as the initial stress increased. The rate of elastic relaxation became slower as the initial stress increased due to the stress dependence of the elastic modulus, as shown in Figure 14. These results show that the initial stress can be estimated from the above factors.

### 3.2. Strain Change Measured by ESPI

Figure 15a,b show typical fringe patterns measured by the 2D-ESPI. The initial stress was 120 MPa. The left images show the carrier fringes introduced before the heating. The fringe intervals became narrower toward the center of the heating point, as shown in the right images, indicating that thermal expansion occurred.

**Figure 16:**
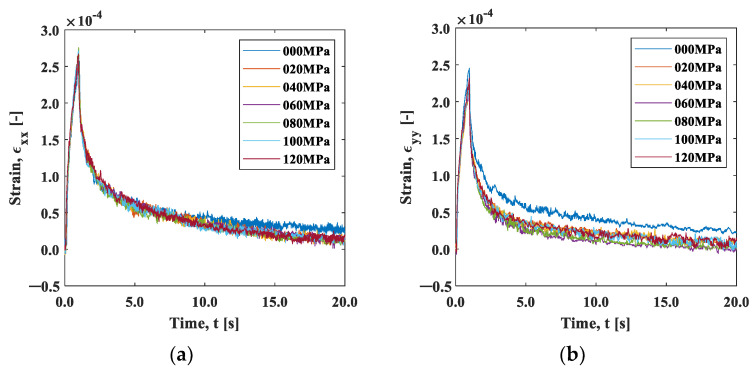
Strain–time curve obtained using the ESPI: (**a**) x-direction; and (**b**) y -direction.

Figure 16 shows the time variation of the mean strains in area A. The strain–time curve exhibits almost the same pattern as that obtained in the strain gauge measurement; the thermal strain increased instantaneously, followed by an exponential decrease in the strain. On the other hand, the strain value was slightly larger than that measured with the strain gauge. The ESPI can directly measure the strain field of the heating point, while the strain gauge shows the averaged strain value in the gauge length that is applied around the heating point. This may result in a larger value being returned by the ESPI. In addition, the strain value measured with the ESPI includes higher noise. This temporal noise may depend on the interval of the carrier fringe initially introduced. In this experiment, the strain was calculated from the spatial frequency (number of fringes in the region of interest) of the carrier fringes. The ratio of the fringe number before/after the deformation became larger as the number of fringes decreased, leading to high sensitivity for displacement, while the analysis was affected by thermal fluctuation and mechanical vibration. In addition, the resolution of the analysis had a limitation due to the pixel resolution of the CMOS camera. The resultant data contain strain noise on the order of 10^−5^. Although this noise level is greater than that of the strain gauge, Figure 16 indicates that the S/N (signal-to-noise) ratio was satisfactorily small.

**Figure 17:**
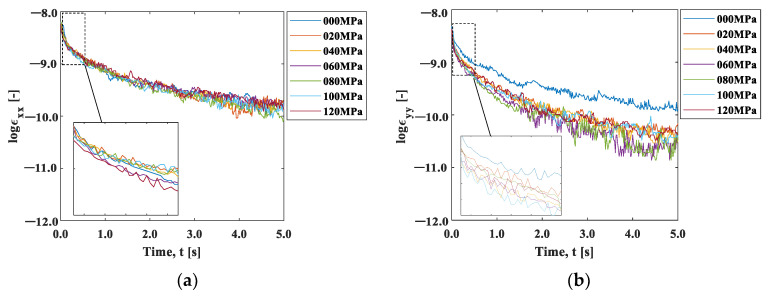
Time–log (strain) curve obtained using the ESPI: (**a**) x-direction; and (**b**) y -direction.

**Figure 18:**
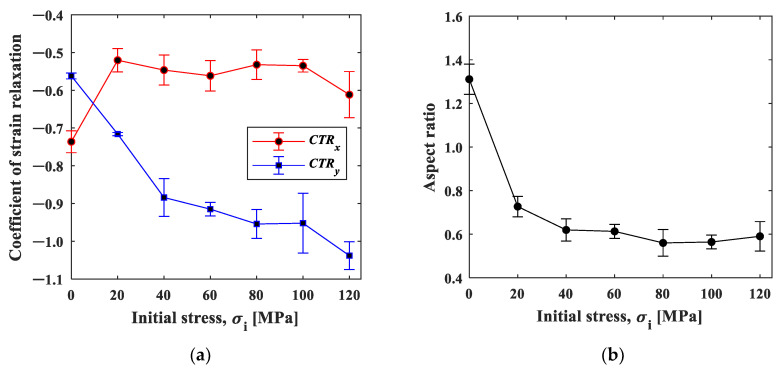
(**a**) CSR–initial stress curve; and (**b**) aspect ratio.

**Figure 19:**
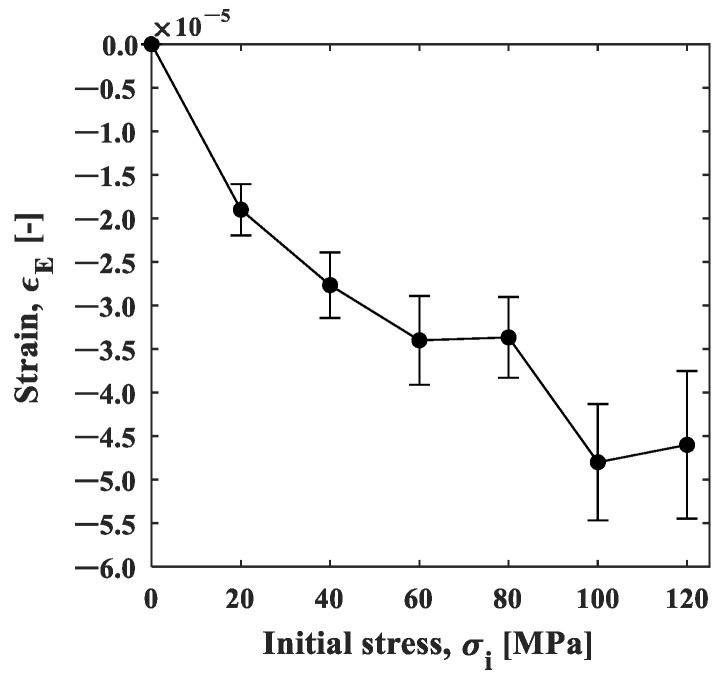
Strain–initial stress plot for area B obtained by the ESPI measurement.

Here, the stress dependences can be discussed in the same manner as the strain gauge measurement. Figure 17 shows the natural logarithm of the strain. The first-order approximation of the curve for 0.4 s after heating and the slope for each stress are shown in Figure 18. The strain relaxation coefficient obtained by the ESPI measurement showed stress dependency, similar to the strain gauge measurement; the *CSR* in the y-direction decreased with the initial stress, and the *CSR* in the x-direction and its aspect ratio also showed the same tendency as the strain gauge measurement. Furthermore, the maximum strain in the unheated area (area B) during the local heating test was plotted against the initial stress, as shown in Figure 19. It is confirmed that the nonthermal strain relaxation increased with increasing stress, although there was some scatter. The above results show that the ESPI allows the same residual stress analysis as the strain gauge measurement.

**Figure 20:**
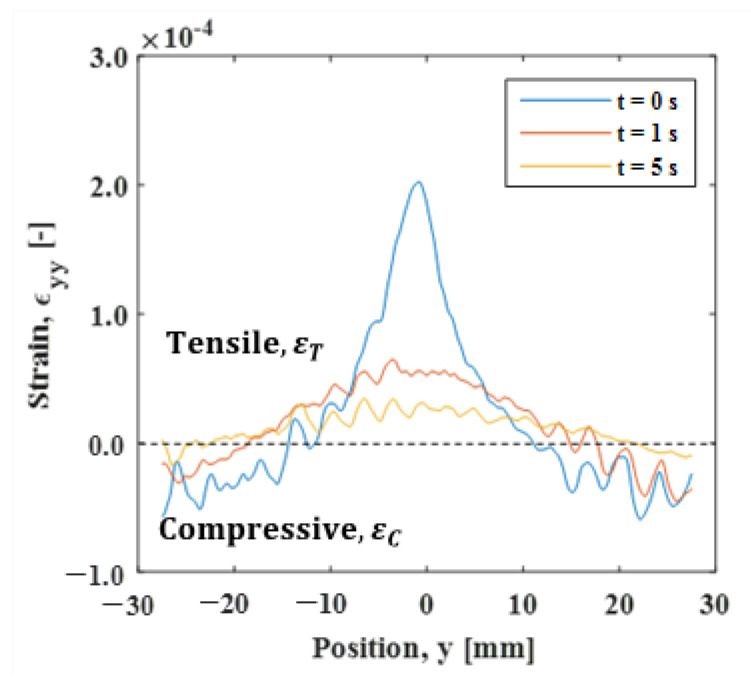
Strain distribution in the y-direction.

**Figure 21:**
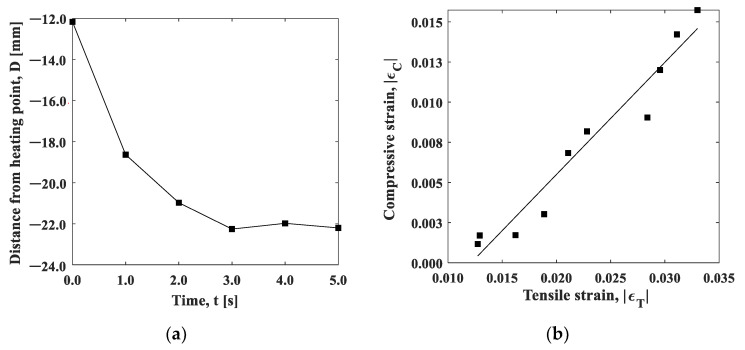
(**a)** Boundary position between compression and tension; and (**b**) relationship between $\left|\varepsilon_{T}\right|$ and $\left|\varepsilon_{C}\right|$.

The full-field measurement can be performed using an ESPI. Figure 20 shows the spatial strain distribution in the y-direction measured during the cooling process. The initial stress $\sigma_{i}$ was 120 MPa. The strain exhibited a Gaussian distribution at *t* = 0 s (immediately after the heating stopped), while the width of the function was larger than the temperature distribution shown in Figure 4. This also indicates that the strain relaxation occurred in a different manner to the thermal diffusion. As the distance from the heat spot increased, the strain decreased towards the negative range, indicating compressive deformation. In this study, the strain distribution was obtained through the frequency analysis of the carrier fringe introduced before the heating. The strain fluctuation observed in the region where the strain was close to zero was due to the fact that, in this region, the high fringe density compromises the spatial resolution of the image. As revealed in the strain gauge measurements, the relaxation of tensile strain in the heated area and the compressive strain in the non-heated area occurred simultaneously during the experiment. The boundary of tension/compression in the strain profile may provide an indication of the position where the strain was balanced. At the cooling time of 1s, the strain exhibited tensile strain at a distance of about 10 mm from the heated area, above which it became compressive. The time variation of the boundary position between $\varepsilon_{T}$ and $\varepsilon_{C}$ is shown in Figure 21a. With the elapse of time, the boundary position moved away from the heated area, converging to −22.0 mm at *t* = 3.0. The strain relaxation induced by the removal of thermal expansion propagated in this time. Furthermore, area B, which was 25 mm away from the heat spot, can be regarded as the area that was not affected by the heat conduction. The relationship between $\left|\varepsilon_{T}\right|$ and $\;\left|\varepsilon_{C}\right|$ showed a linear relation, implying that $\Delta \varepsilon_{T}/\Delta \varepsilon_{C}≃const$. This fact directly indicates that the strain in the heat spot was constantly balanced with the nonthermal strain in the less heat-affected area, regardless of the thermal diffusion. 

## 4. Conclusions

The present study investigated the effect of the stress condition on the thermal deformation behavior through local spot heating in the temperature range of room temperature to approximately +10 °C. The strain was measured using a strain gauge in a contact way, and a two-dimensional ESPI in a non-contact way. The following results were obtained:

(1) Relaxation of the positive (tensile) strain in the local spot-heated area occurred more slowly than the thermal relaxation due to the heat diffusion, and it showed an exponential decay behavior.
(2) The coefficient of strain relaxation obtained by the strain relaxation curve in the shorter time depended on the tensile stress initially applied, which was attributed to the stress dependency of elasticity.
(3) In the less heat-affected area (the area far from the heated area), the compressive strain was induced by the thermal expansion of the heated area. The compressive strain in the cooling process also showed stress dependency.
(4) The two-dimensional ESPI allowed the visualization of the above strain relaxation behavior in a non-contact way. These results indicate the feasibility of non-destructive and non-contact residual stress estimation through the evaluation of the above relaxation coefficients.

## Figures and Tables

**Table 1:** Chemical composition of AA5083 (mass %).

Mg	Si	Fe	Cu	Mn	Cr	Zn	Ti	Al
4.0–4.9	<0.4	<0.4	<0.1	0.4–4.0	0.05–0.25	<0.25	<0.15	Bal.

## Data Availability

Not applicable.
